# Comparative Transcriptomic and Proteomic Analyses Identify Key Genes Associated With Milk Fat Traits in Chinese Holstein Cows

**DOI:** 10.3389/fgene.2019.00672

**Published:** 2019-08-13

**Authors:** Chenghao Zhou, Dan Shen, Cong Li, Wentao Cai, Shuli Liu, Hongwei Yin, Shaolei Shi, Mingyue Cao, Shengli Zhang

**Affiliations:** Key Laboratory of Animal Genetics, Breeding and Reproduction, Ministry of Agriculture & National Engineering Laboratory for Animal Breeding, College of Animal Science and Technology, China Agricultural University, Beijing, China

**Keywords:** milk fat, transcriptomic, proteomic, Chinese Holstein, liver

## Abstract

Milk fat is the most important energy substance in milk and contributes to its quality and health benefits. However, the genetic mechanisms underlying milk fat synthesis are not fully understood. The development of RNA sequencing and tandem mass tag technologies has facilitated the identification of eukaryotic genes associated with complex traits. In this study, we used these methods to obtain liver transcriptomic and proteomic profiles of Chinese Holstein cows (*n* = 6). Comparative analyses of cows with extremely high vs. low milk fat percentage phenotypes yielded 321 differentially expressed genes (DEGs) and 76 differentially expressed proteins (DEPs). Functional annotation of these DEGs and DEPs revealed 26 genes that were predicted to influence lipid metabolism through insulin, phosphatidylinositol 3-kinase/Akt, mitogen-activated protein kinase, 5′ AMP-activated protein kinase, mammalian target of rapamycin, and peroxisome proliferator-activated receptor signaling pathways; these genes are considered as the most promising candidate regulators of milk fat synthesis. The findings of this study enhance the understanding of the genetic basis and molecular mechanisms of milk fat synthesis, which could lead to the development of cow breeds that produce milk with higher nutritional value.

## Introduction

Milk products are an important part of our daily diet. There are a multitude of different milk products that vary in terms of composition, including fatty acid and protein content. Milk contains approximately 3–5% fat, which is the most important energy-rich substance it contains. The nutritional value of milk fat depends on the composition of fatty acids (FAs), which are classified according to hydrocarbon chain length as short-chain (C4–C10), medium-chain (C11–C17), and long-chain (LC, ≥C18) FAs, and according to the degree of saturation of the hydrocarbon chains as saturated (S)FAs, monounsaturated FAs, and polyunsaturated (PU)FAs. High concentrations of SFAs such as myristic acid (C14:0), lauric acid (C12:0), and palmitic acid (C16:0) increase low-density lipoprotein (LDL) concentration in the blood, which has been linked to cardiovascular and cerebrovascular diseases (Mensink et al., 2003). Meanwhile, PUFAs such as conjugated and unconjugated linoleic acid (C18:2) play a beneficial role in reducing blood lipids, suppressing the immune response, promoting bone formation, and stimulating lipid metabolism (Belury, 2002). The ratio of PUFA to SFA is an important indicator of diet quality. The main proteins in milk are α_s1_-casein (CN), α_s2_-CN, β-CN, κ-CN, α-lactalbumin, and β-lactoglobulin, which are known to contribute to lipid synthesis and metabolism in humans (Mcgregor and Poppitt, 2013). The liver is a complex digestive gland in ruminant animals, including dairy cattle, and plays an important role in the metabolism of carbohydrates, fats, proteins, vitamins, hormones, and other substances. Nutrients absorbed from the digestive tract pass through the liver, enter the circulatory system, and finally arrive in the mammary glands of dairy cattle. The liver thus plays a critical role during lactation in cattle (Dorland et al., 2009; Graber et al., 2010; Schlegel et al., 2012).

There are few reports on the breeding of transgenic dairy cows, and cows that produce low-fat, high-protein milk have not been developed to date. The main constraint is the difficulty in obtaining animals that are true-breeding for this particular trait owing to the lack of information on related genes. The 29 autosomes of cows harbor most of the genes controlling milk traits and production (e.g., fat and protein content), including diacylglycerol O-acyltransferase (*DGAT*)*1* p.Lys232Ala and stearoyl-coenzyme (Co)A desaturase1 p.Ala293Val (Mele et al., 2007; Schennink et al., 2008; Conte et al., 2010), and many important or suggestive genomic regions have been identified (Schennink et al., 2009b; Stoop et al., 2009).

The development of RNA sequencing (RNA-seq) and tandem mass tag (TMT) technologies has enabled the identification of eukaryotic genes associated with complex traits through analysis of transcriptomic and proteomic profiles with low bias, broad dynamic range, low rate of false positive signals, and high reproducibility. The TMT method employs a set of amine-reactive isobaric tags to derivatize peptides at the N terminus and at lysine side chains, thereby facilitating simultaneous protein identification and quantification *via* mass spectrometry (MS) analysis of peptide fragments. Both RNA-seq and TMT have been widely used to screen for functional genes associated with milk composition in cattle and other domestic animals. In the present study, we compared the liver transcriptome and proteome profiles of Chinese Holstein cows with extremely high and low phenotypic values for milk fat and identified genes and proteins involved in milk production.

## Materials and Methods

### Sample Collection

Based on milk production in their previous lactation, six Chinese Holstein cows—of which three were in their second and three in their third lactation—were selected from the Beijing Sanyuan Lvhe Dairy Farm and divided into high milk fat percentage (HP) and low milk fat percentage (LP) groups, each with three cows. The average milk fat percentage in this population was 3.7% (2.3–3.9%). Based on Dairy Herd Improvement system (DHI) data, we defined a high milk fat percentage group as those cows with 3.7% milk fat, and the low milk fat percentage group was composed of cows with 3.2% milk fat. The phenotype information of six Chinese Holstein cattle are showed in **Table S1**. The cows were kept in free stall housing, fed a total mixed ration (TMR, containing 16.1% crude protein, 22.9% acid detergent fiber), and had access to water ad libitum. Cows were milked three times daily in the milking parlor. The age differences among the cows in the second lactation were less than 45 days. Among the six cows, there were two pairs of half sibs consisting of one HP and one LP cow; the other two cows—one HP and one LP cow—were non‐sibs. The cows were killed by electroshock, bled, skinned, and dismembered in the same slaughterhouse. Liver tissue samples (approximately 0.5–1. 0g) from each individual were removed within 30 min after slaughter. Five pieces of liver tissue samples per cow were carefully collected for RNA isolation, placed into a clean RNAse-free Eppendorf tube, and stored in liquid nitrogen. All sample collection procedures were carried out in strict accordance with the protocol approved by the Animal Welfare Committee of China Agricultural University (Permit Number: DK996).

### RNA Isolation, Library Preparation, and Sequencing

Total RNA was extracted from the bovine liver tissue using the Trizol method (Invitrogen, Carlsbad, CA) according to the manufacturer’s instructions. RNA degradation and contamination was monitored on 1% agarose gels. RNA concentration was measured using a NanoDrop 2000 spectrophotometer (Thermo Fisher Scientific, Waltham, MA, USA). RNA integrity was assessed using the RNA Nano 6000 Assay Kit of the Agilent Bioanalyzer 2100 system (Agilent Technologies, Santa Clara, CA, USA). The six purified RNA samples had an RIN ≥ 7.0, and a total of 1 μg RNA per sample was used as input material for RNA sample preparation. Sequencing libraries were generated using the NEBNext Ultra RNA Library Prep Kit for Illumina (New England Biolabs, Ipswich, MA, USA) according to the manufacturer’s recommendations, and index codes were added to attribute sequences to each sample. Briefly, mRNA was purified from total RNA using poly-T oligo-attached magnetic beads. Fragmentation was carried out using divalent cations under elevated temperature in NEBNext First Strand Synthesis Reaction Buffer (5×). First strand cDNA was synthesized using random hexamer primer and Moloney murine leukemia virus reverse transcriptase; second strand cDNA synthesis was then performed using DNA polymerase I and RNase H. Remaining overhangs were converted into blunt ends through exonuclease/polymerase activities. After adenylation of 3′ ends of DNA fragments, NEBNext Adaptor with a hairpin loop structure was ligated to prepare the fragments for hybridization. In order to select cDNA fragments with a length of approximately 240 bp, the library fragments were purified with the AMPure XP system (Beckman Coulter, Beverly, MA, USA). A 3-μl volume of USER Enzyme (New England Biolabs) was incubated with size-selected, adaptor-ligated cDNA at 37°C for 15 min followed by 5 min at 95°C. PCR was performed with Phusion High-Fidelity DNA polymerase, universal PCR primers, and index (X) primer. PCR products were purified (AMPure XP system), and library quality was assessed with the Agilent Bioanalyzer 2100. Index-coded samples were clustered on a cBot Cluster Generation System using TruSeq PE Cluster Kit v.4-cBot-HS (Illumina, San Diego, CA, USA) according to the manufacturer’s instructions. After cluster generation, cDNA libraries were sequenced on an Illumina platform, and paired-end reads were generated.

### Mapping and Annotation of Sequencing Reads

Raw data (raw reads) in fastq format were first processed with in-house Perl scripts. In this step, clean data (reads) were obtained by removing those containing adapter and poly-N sequences and low-quality reads. At the same time, Q20, Q30, GC content, and sequence duplication level of the clean data were calculated. All downstream analyses were based on high-quality clean data; these reads were mapped to the reference genome sequence (UMD3.1.80). Only reads with a perfect match or one mismatch were further analyzed and annotated based on the reference genome. HISAT2 (https://ccb.jhu.edu/software/hisat2/index.shtml) was used for mapping to the reference genome.

### Quantification and Differential Gene Analysis by RNA-seq

Fragments per kilobase of exon per 106 mapped fragments (FPKM) values obtained using Cufflink v.2.1.1 software (http://cole-trapnell-lab.github.io/cufflinks/) were used as values for normalized gene expression. Differential expression analyses of HP vs. LP were performed using DESeq2 (Love et al., 2014), which provides statistical tools for identifying differential expression in digital gene expression data using a model based on the negative binomial distribution. The resultant *p* values were adjusted using Hochberg method for controlling the false discovery rate (FDR). *q* value < 0.01 and | log2 [fold change (FC)]| ≥ 1 were set as thresholds for significantly different expression.

### Protein Isolation, Enzymolysis, and TMT Labeling

The 500 μl SDT buffer was added to the 50 mg samples, which were transferred to 2-ml tubes containing quartz sand (with 1/4-inch ceramic beads included for tissue samples). The lysate was homogenized twice for 60 s each (24 × 2, 6.0 m/s) with a homogenizer (MP Biomedicals, Solon, OH, USA). The homogenate was boiled for 3 min and then sonicated for 2 min. After centrifugation at 20,000 × *g* for 20 min at 4°C, the concentration of proteins in the filtrate was quantified with a BCA Protein Assay Kit (Bio-Rad, Hercules, CA, USA). DTT and UA buffer (8 M Urea, 150 mM Tris-HCl, pH 8.0) were added to 300 μg of the supernatant and the resulting mix was passed through a 10 KD filter. The protein samples were centrifuged with UA buffer, IAA (50mM IAA in UA), and NH_4_HCO_3_ buffer and then treated overnight with trypsin at a trypsin-to-protein ratio of 1:100. The peptide mixture (100 μg) of each sample was labeled using 10PLEX TMT reagent according to the manufacturer’s instructions (Thermo Fisher Scientific).

The peptide mixture was loaded onto a reversed-phase trap column (Thermo Scientific Acclaim PepMap100, 100 μm × 2 cm, nanoViper C18) connected to a C18 reversed-phase analytical column (length = 10 cm, inner diameter = 75-μm, 3-μm resin; Thermo Fisher Scientific) in buffer A (0.1% formic acid) and separated for 1.5 h with a linear gradient of buffer B (98% acetonitrile and 0.1% formic acid) at a flow rate of 300 nL/min controlled by IntelliFlow technology (4%–7% buffer B for 2 min, 7%–20% buffer B for 65 min, 20%–35% buffer B for 12 min, 35%–90% buffer B for 2 min, and holding in 90% buffer B for 9 min).

### Liquid Chromatography Tandem MS (LC-MS/MS) Analysis

LC-MS/MS analysis was performed on a Q-exactive Plus Orbitrap mass spectrometer (Thermo Fisher Scientific) coupled to an Easy nLC chromatograph (Proxeon Biosystems, now Thermo Fisher Scientific) for 90 min. The instrument was operated in positive ion mode. MS data were acquired using a data-dependent top 10 method to dynamically select the most abundant precursor ions from the survey scan (300–1,800 m/z) for higher-energy collisional dissociation (HCD) fragmentation. The automatic gain control target was set to 1e6, with a maximum injection time of 50 ms. The duration of dynamic exclusion was 40.0 s. Survey scans were acquired at a resolution of 70,000 at m/z 200, and resolution for HCD spectra was set to 35,000 at m/z 200 (TMT 10PLEX), with an isolation window of 1.6 Th. Normalized collision energy was 35 eV, and the underfill ratio—which specifies the minimum percentage of the target value likely to be reached at maximum fill time—was defined as 0.1%. The instrument was run with the peptide recognition mode enabled.

### Database Search and Protein Identification and Quantification

For peptide identification and quantification, MS/MS data were searched against the “Uniprot-Bos taurus_32310_20180905.fasta” file using Maxquant version 1.6.0.16. The following parameters were used: trypsin as enzyme specificity; maximum two missed cleavages permitted; fixed modification: carbamidomethylation of cysteine residues; variable modifications: oxidation of methionine residues and N-terminal acetylation; first search peptide tolerance of 20 ppm; main search peptide tolerance of 4.5 ppm. Protein quantification was based on the razor and unique peptides. Fold decrease/increase >1.2 and *p* < 0.05 were set as the threshold for identifying differentially expressed proteins (DEPs).

### Gene Ontology (GO) Enrichment Analysis and Kyoto Encyclopedia of Genes and Genomes (KEGG) Pathway Enrichment Analysis

GO enrichment analysis of differentially expressed genes (DEGs) was performed with the GOseq R packages based on a Wallenius non-central hyper-geometric distribution (Young et al., 2010), which can adjust for gene length bias. The KEGG database (Kanehisa et al., 2007) is used to analyze high-level functions of a biological system based on molecular-level information, especially large-scale molecular datasets generated by genome sequencing and other high-throughput approaches (http://www.genome.jp/kegg/). We used KOBAS software (Mao et al., 2005) to assess the enrichment of DEGs in KEGG pathways.

### Protein–Protein Interactions (PPIs) and Mapping of Quantitative Trait Loci (QTL)

The sequences of DEGs were searched against the genome of a related species using blastx; we searched the STRING database (http://string-db.org/) to determine the predicted PPIs of these DEGs. The PPIs were visualized using Cytoscape (Shannon et al., 2003). We also integrated the DEGs and QTL for milk fat traits from the QTLdb database (http://www.animalgenome.org/cgi-bin/QTLdb/BT/index) into our analysis.

### Verification of RNA-seq and TMT Data

RT-qPCR primers were designed to span the exon-exon boundaries of eight genes selected by RNA-seq; RT-PCR analysis was performed using a SYBR^®^ Premix Ex Taq™ II (Tli RNaseH Plus), ROX plus (RR82LR, TaKaRa) on a ABI7500 Real-Time PCR Detection System (Applied Biosystems), according to the manufacturer’s instructions.

The protein expression levels obtained using TMT analysis were confirmed by quantifying the expression levels of five selected proteins by a parallel reaction monitoring (PRM) analysis carried out at the Beijing Bangfei Bioscience Co., Ltd. (Beijing, China). PRM is a targeted method of quantification performed using high-resolution hybrid mass spectrometers such as quadrupole-Orbitrap (q-OT). Signature peptides for the target proteins were defined according to the TMT data, and only unique peptide sequences were selected for the PRM analysis. Each protein sample (50 μg) was separated using a nanoliter flow HPLC liquid phase system Easy nLC 1200 (Thermo Fisher). Samples were loaded by an autosampler into a mass spectrometer pre-column C18 trap column (C18, 3 μm, 100 μm × 20 mm) and separated by an analytical column C18 column (C18, 3 μm, 75 μm × 150 mm). After peptide separation, targeted PRM mass spectrometry was performed using a Q-Exactive Plus mass spectrometer (Thermo Scientific). The result of mass spectrum was analyzed using the software Skyline 4.1.

## Results

### Overview of RNA Transcriptomic Profiles of Cow Liver Tissue

A total of 197,358,565 paired-end reads were obtained by RNA-seq. The quality value of Q30 for sequencing was no less than 96.40% for each sample. An average of 91.55% (range: 93.87%–95.31%) of reads were mapped to the bovine genome (Ensembl UMD3.1) using HISAT2. Of these, approximately 91.42% (range: 90.75%–92.10%) were uniquely mapped and 3.29% (range: 3.07%–3.84%) were multi-mapped reads (**Table S2**). Additionally, of the total mapped reads, roughly 70% in each group corresponded to exons (**Figure S1**).

### Analysis of DEGs

**Figure 1:**
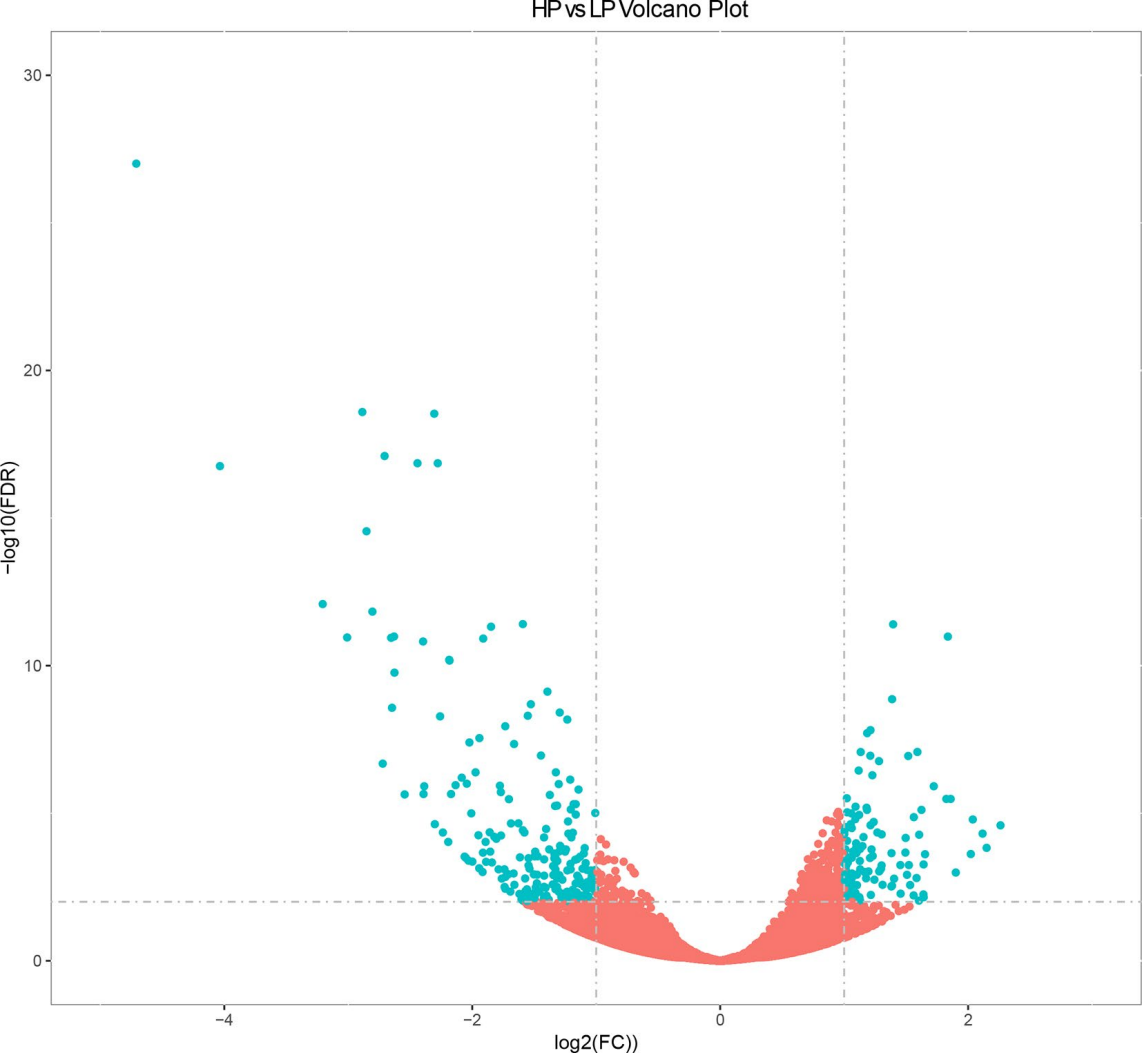
Volcano plot displaying differential expressed genes in bovine liver tissues with transcriptomic analyses within two different comparison groups. The *y* axis corresponds to the mean expression value of log10 (*q* value), and the *x* axis displays the log2 fold change value. The blue dots represent the significantly differential expressed transcripts; the red dots represent the transcripts whose expression levels did not reach statistical significance.

The expression levels of known and novel genes were calculated as FPKM using DESeq2 (Love et al., 2014), which provides statistical approaches for identifying differentially expressed known and novel genes based on a negative binomial distribution model. A total of 23,098 genes were expressed in liver tissue. Pairwise comparisons according to stringent criteria—i.e., | log2 (FC) | > 2 and *q* < 0.01—were carried out to identify DEGs (**Figure 1**). A total of 321 genes were differentially expressed between HP and LP groups, including 117 that were up-regulated and 204 that were down-regulated (**Table S3** and **Figure 1**, *q* value < 0.01). The results of cluster analysis of DEGs are depicted in a heatmap (**Figure S2**).

### Functional Analysis of DEGs

**Figure 2:**
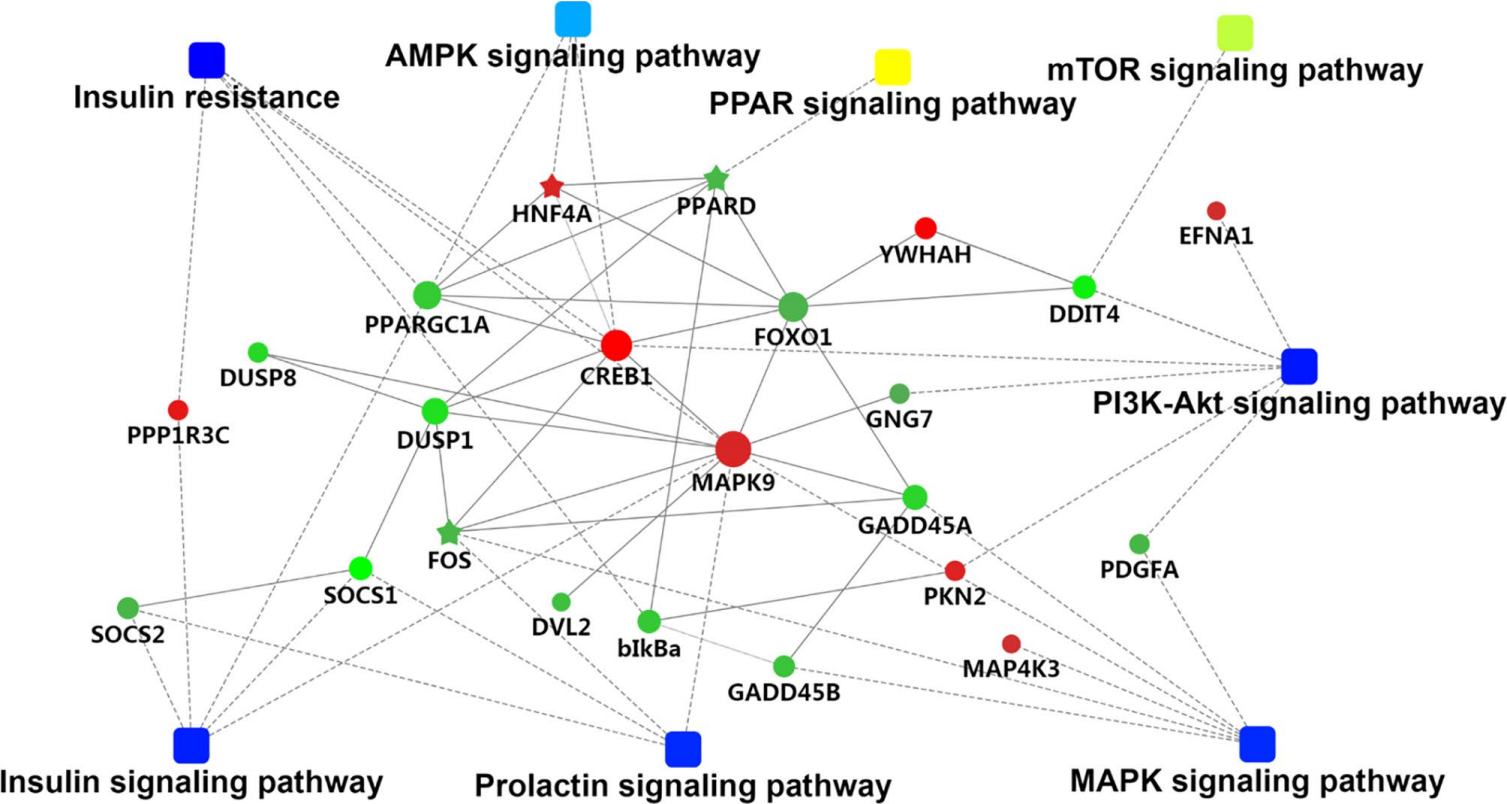
The metabolic network comprising candidate genes in protein–protein interactions (PPI) network and pathways with transcriptomic analyses. The round nodes indicate genes, red indicates up-regulation, and green indicates down-regulation. The rectangular node represents the KEGG pathway/biological process, and the significant *p* value is represented by yellow-blue gradient; yellow indicates a small *p* value, while blue indicates a large *p* value.

We used GOseqR packages and the KEGG database to determine the function of the identified DEGs. The top three functions related to metabolism were “cell adhesive protein binding involved in bundle of His cell-Purkinje myocyte communication,” “polyamine oxidase activity,” and “serine and oxidoreductase activity, acting on the CH-NH group of donors, oxygen as acceptor” (KS ≤ 1.0E−30) (**Table S4**). We identified a metabolic network comprising 22 DEGs involved in insulin production (mitogen-activated protein kinase [*MAPK*]*9*, cyclic AMP response element-binding protein [*CREB*]*1*, protein phosphatase 1 regulatory subunit [*PPP1R*]*3C*, nuclear factor κB inhibitor α [*NFKBIA*], peroxisome proliferator-activated receptor γ, coactivator 1α [*PPARGC1A*], and forkhead box [*FOX*]*O1*); insulin resistance (*MAPK9*, *CREB1*, *PPP1R3C*, *NFKBIA*, *PPARGC1A*, and *FOXO1*); phosphatidylinositol 3-kinase (PI3K)/Akt signaling (DNA damage-inducible transcript [*DDIT*]*4*, *CREB1*, G protein subunit γ [*GNG*]*7*, platelet-derived growth factor subunit [*PDGF*]*A*, ephrin A1, protein kinase [*PKN*]*2*, breast cancer type 1 susceptibility gene, and tyrosine 3-monooxygenase/tryptophan 5-monooxygenase activation protein η); MAPK signaling (FBJ murine osteosarcoma viral oncogene homolog[*FOS*], *MAPK9*, growth arrest and DNA damage-inducible α [*GADD*]*45A*, dual specificity phosphatas*e* [*DUSP*]*1*, platelet-derived growth factor subunit A [*PDGFA*], *MAP4K3*, *GADD45B*, and *DUSP8*); prolactin production (*FOS*, suppressor of cytokine signaling [*SOCS*]*1*, *MAPK9*, *CREB1*, and *SOCS2*); 5′ AMP-activated protein kinase (AMPK) signaling (hepatocyte nuclear factor 4α [*HNF*]*4A*, *PPARGC1A*, and *FOXO1*); mammalian target of rapamycin (mTOR) signaling (DNA damage inducible transcript [*DDIT*]*4* and disheveled segment polarity protein 2); and PPAR signaling (*PPARδ* [*PPARD*]) (**Table S5** and **Figure 2**). The FOXO and CREB families have key features for the integration of insulin production and insulin resistance signaling with glucose and lipid metabolism (Lee and Dong, 2017). The PI3K/AKT signaling pathway played a key role in regulating lipid metabolism in lactating goats (Li et al., 2018). The results of a previous GWAS study showed that the MAPK signaling pathway was overrepresented for milk protein and fat content (Cecchinato et al., 2019). Prolactin production, AMPK, and PPAR signaling pathways are well known for regulating milk fat synthesis (Schennink et al., 2009a; Liu et al., 2016; Gao et al., 2017). These networks play critical roles in the regulation of milk fat synthesis (Anderson et al., 2007; Bionaz and Loor, 2011). On the basis of their biological function and PPI analysis, 16 of the 22 genes were considered as important for lipid metabolism in the liver (**Table 1**).

**Table 1:** Expression changes of the candidate genes in bovine liver tissue with transcriptomic analyses.

Symbol	Chromosome	Gene name	HP counts	LP counts	Log2 fold change	*Q*-value
*SOCS1*	25	Suppressor of cytokine signaling 1	163	2775	−3.0090	1.11E-11
*SLCO4A1*	13	Solute carrier organic anion transporter family member 4A1	2616	21071	−2.7070	7.91E-18
*DDIT4*	28	DNA damage inducible transcript 4	1204	11321	−2.6543	1.14E-11
*MTHFR*	16	Methylenetetrahydrofolate reductase	6728	36841	−2.1832	6.73E-11
*GADD45A*	3	Growth arrest and DNA damage inducible alpha	2506	14092	−2.0076	1.01E-05
*PPARGC1A*	6	PPARG coactivator 1 alpha	1103	4745	−1.7676	5.69E-05
*PDGFA*	25	Platelet derived growth factor subunit A	55	158	−1.3385	6.25E-04
*SOCS2*	5	Suppressor of cytokine signaling 2	596	1650	−1.3137	4.15E-04
*FOXO1*	12	Forkhead box O1	1708	4036	−1.2051	7.46E-06
*SLC22A1*	9	Solute carrier family 22 member 1	18901	47883	−1.1927	7.20E-03
*MAPK9*	7	Mitogen-activated protein kinase 9	1898	788	1.1277	1.63E-04
*HNF4A*	13	Hepatocyte nuclear factor 4 alpha	11382	4804	1.1560	6.41E-05
*CREB1*	2	cAMP responsive element binding protein 1	667	175	1.6049	5.35E-05
*SYBU*	14	Syntabulin	2034	560	1.6236	7.72E-06
*HNF4G*	14	Hepatocyte nuclear factor 4 gamma	2881	642	1.8243	3.28E-06

### Protein Identification and Quantification by TMT

A total of 112,916 spectra were obtained in the 10PLEX LC-MS/MS analysis. After pooling samples from the two groups, 31,327 unique peptides were identified, including 4,356 proteins that were originally identified with the Q-exactive Plus Orbitrap mass spectrometer (**Figure S3a**). To eliminate false positives, we controlled FDR to 1% at both the peptide and protein levels using the MaxQuant reversed sequence database. The number of proteins identified at various molecular weight ranges were as follows: 0–50 kDa, 2,349; 50–100 kDa, 1,215; 100–150 kDa, 350; 150–200 kDa, 100; 200–300 kDa, 86; and 300–3850 kDa, 36. Collectively, these 4,136 proteins accounted for 94.95% of those identified (**Figure S3b**). In addition, most proteins had high peptide coverage; 85.61% and 14.39% had <50% and >50% sequence coverage, respectively (**Figure S3c**). Among the identified proteins, 48.39% were represented by fewer than five peptides (**Figure S3d**), indicating good sequence coverage. Information on the identification of proteins is shown in **Supplementary Tables S6**, **S7**.

### Analysis of DEPs

**Figure 3:**
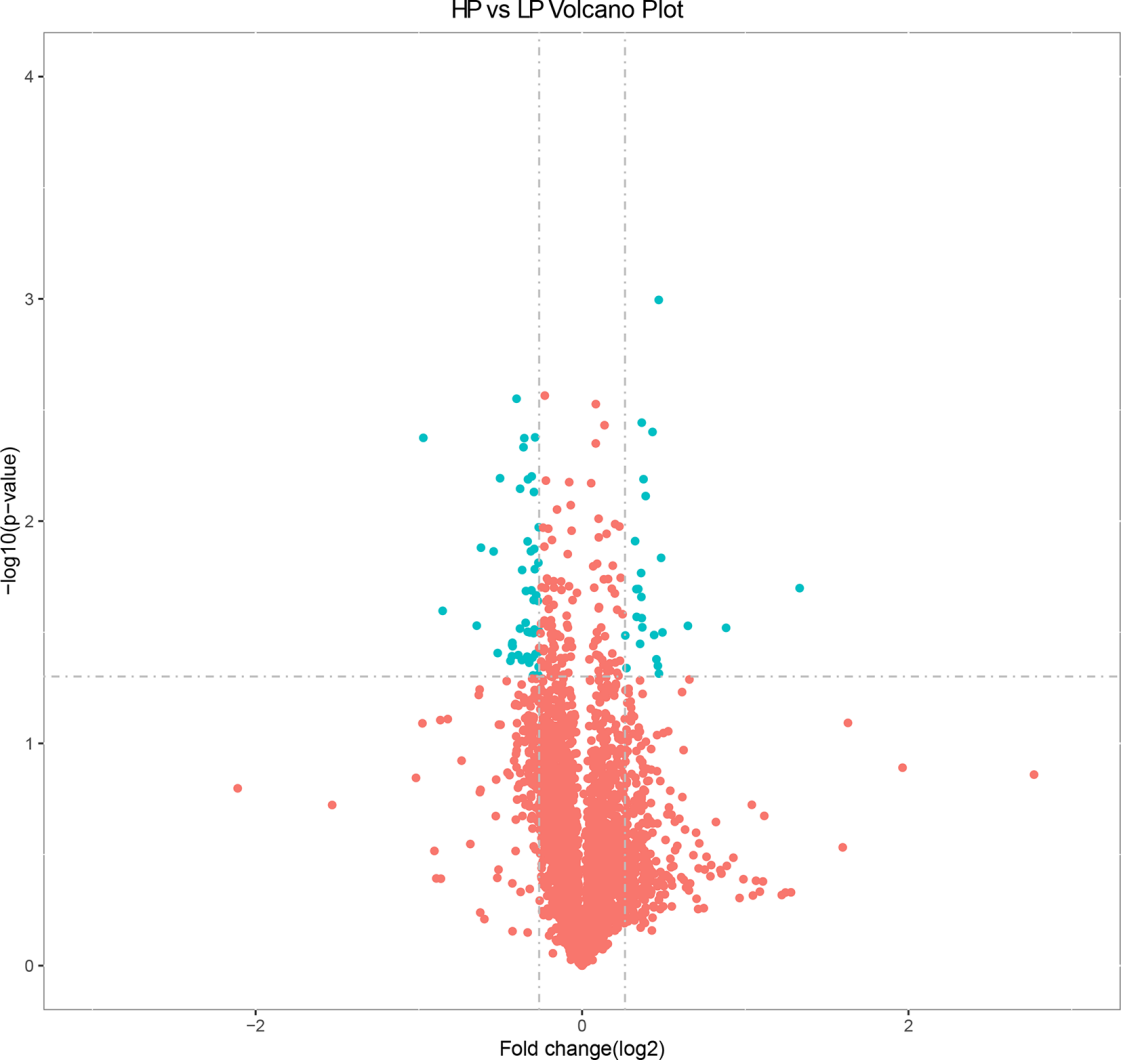
Volcano plot displaying differential expressed proteins in bovine liver tissues with proteomic analyses within two different comparison groups. The *y* axis corresponds to the mean expression value of log10 (*p* value), and the *x* axis displays the log2 fold change value. The blue dots represent the significantly differential expressed transcripts; the red dots represent the transcripts whose expression levels did not reach statistical significance.

Based on the selection criteria (fold decrease/increase >1.2 and *p* < 0.05), we identified 76 DEPs in the HP vs. LP comparisons, including 25 up-regulated and 51 down-regulated DEPs (**Table S7** and **Figure 3**). Clusters of all DEPs were visualized by a heatmap (**Figure S4**).

### Functional Analysis of DEPs

**Figure 4:**
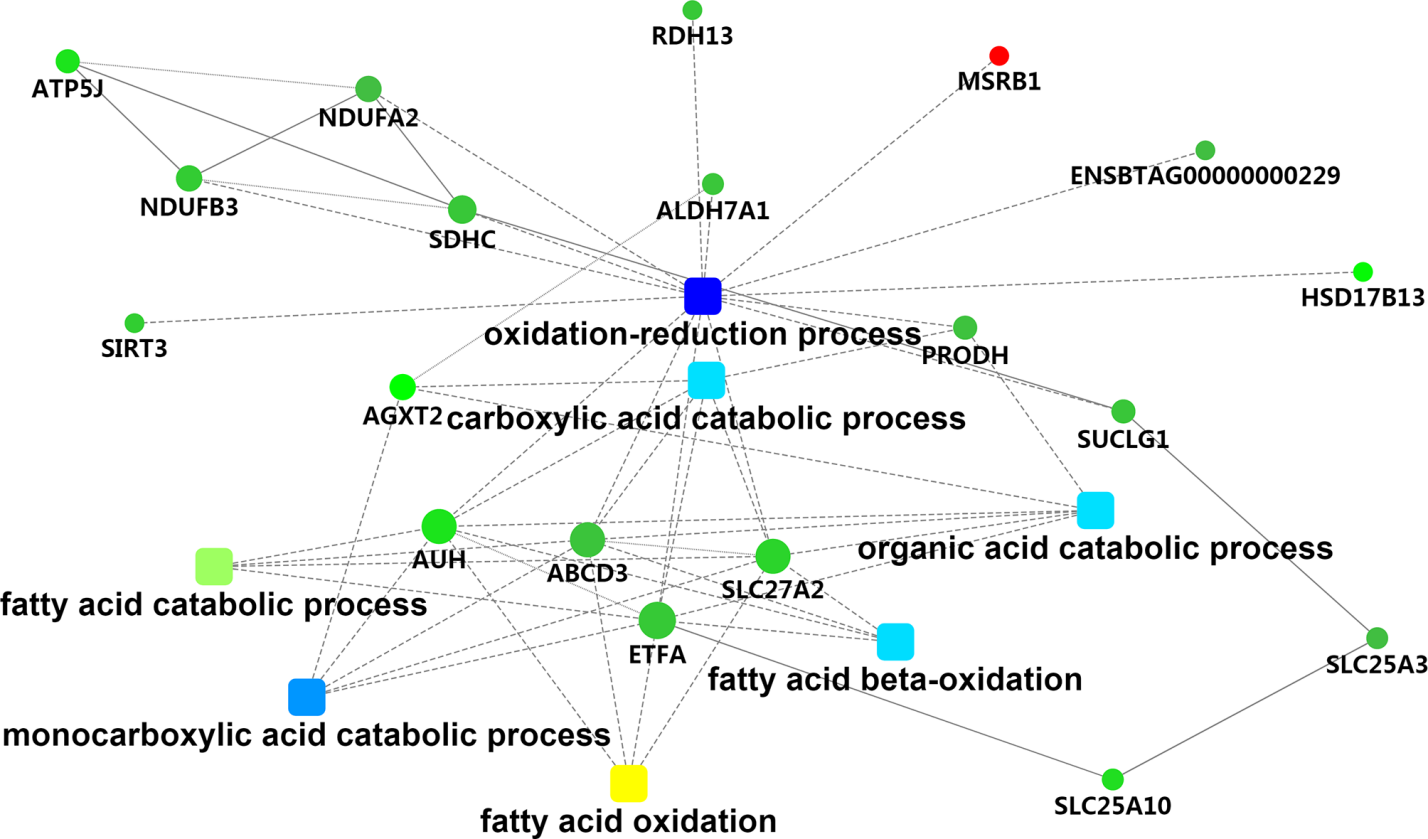
The metabolic network comprising candidate genes in PPI network and GO terms with transcriptomic analyses. Round nodes indicate genes, red indicates up-regulation, and green indicates down-regulation. The rectangular node represents the KEGG pathway/biological process, and the significant *p* value is represented by yellow-blue gradient; yellow indicates a small *p* value, while blue indicates a large *p* value.

To assess the biological significance of these DEPs in hepatic tissue of Holstein cows with different milk fat compositions, the DEPs were further classified based on GO and KEGG functional annotations. For the “cellular component” aspect, the classification analysis revealed that most of the DEPs were related to mitochondria (33.90%), with four related to the endoplasmic reticulum (chloride channel CLIC-like protein [*CLCC*]*1*, heat shock 70 kDa protein [*HSPA*]*13*, transmembrane protein [*TMEM*]*33*, and solute carrier family 27 member 2 [*SLC27A2*]). For “biological process,” the GO terms were mainly associated with “oxidation-reduction process” (methionine-R-sulfoxide reductase [*MSR*]*B1*, ATP binding cassette subfamily D member 3 [*ABCD3*], aldehyde dehydrogenase 7 family member A1 [*ALDH7A1*], AUH protein [*AUH*], SLC27A2, electron transfer flavoprotein subunit alpha [ETFA], ENSBTAG00000000229, proline dehydrogenase 1 [*PRODH*], NAD-dependent protein deacetylase [*SIRT3*], succinate dehydrogenase cytochrome b560 subunit [*SDHC*], retinol dehydrogenase [*RDH*]*13*, hydroxysteroid (17-beta) dehydrogenase [*HSD17B*]*13*, succinate–CoA ligase [ADP/GDP-forming] subunit alpha [*SUCLG1*], NADH dehydrogenase [ubiquinone] 1 [*NDUF*]*B3*, and *NDUFA2*), “monocarboxylic acid catabolic process” (alanine–glyoxylate aminotransferase [*AGXT*]*2*, *ABCD3*, *AUH*, *SLC27A2*, and *ETFA*), “fatty acid beta-oxidation” (*ABCD3*, *AUH*, *SLC27A2*, and *ETFA*), “carboxylic acid catabolic process” (*ABCD3*, *AUH*, *SLC27A2*, *ETFA*, *PRODH*, and *AGXT2*), “fatty acid catabolic process” (*ABCD3*, *AUH*, *SLC27A2*, and *ETFA*), and “very long-chain fatty acid catabolic process” (*ABCD3* and *SLC27A2*). Functional associations among these GO terms were visualized using the STRING database (**Table S8** and **Figure 4**). We also found potentially relevant GO terms in “molecular function,” including “fatty acid transporter activity” (*ABCD3* and *SLC27A2*); “long-chain fatty acid binding” (S100 calcium-binding protein [*S100*]*A9* and *S100A8*); and “oxidoreductase activity” (*MSRB1*, *ALDH7A1*, *ETFA*, *ENSBTAG00000000229*, *PRODH*, *SDHC*, *NDUFA2*, *RDH13*, and *HSD17B13*).

KEGG pathway analysis of the significantly altered proteins revealed 13 enriched canonical pathways (*p* < 0.05) (**Table S9**); the top three related to metabolism were “oxidative phosphorylation,” “citrate cycle and glycine,” and “serine and threonine metabolism” (*p* = 3.11E−04, 2.45E−03, and 4.76E−03, respectively). The major functional associations within these pathways were visualized using the STRING database. Notably, five genes encoding enriched DEPs were related to insulin resistance (*SLC27A2* and phosphoenolpyruvate carboxykinase [*PCK*]*1*), insulin secretion (phosphoinositide phospholipase C-β2 [*PLCB2*]), insulin signaling (*PCK1*), PI3K/Akt signaling (*PCK1*), AMPK signaling (*PCK1*), and PPAR signaling (cytochrome P450 family 4 subfamily A member [*CYP4A*]*11*, *SLC27A2*, carnitine palmitoyltransferase [*CPT*]*2*, and *PCK1*). The details of the five candidate genes with proteomic profiles are shown in **Table 2**.

**Table 2:** Expression changes of the candidate genes in bovine liver tissue with proteomic analyses.

Symbol	Protein ID	Gene name	Chromosome	Sequence length	Log2 fold change	*P*-value
*SLC27A2*	F1MQP2	Solute carrier family 27 (fatty acid transporter), member 2	10	620	−0.3590	0.0047
*PCK1*	F1N1Z7	phosphoenolpyruvate carboxykinase 1	13	622	−0.4296	0.0406
*CPT2*	F1N1M7	carnitine palmitoyltransferase 2	3	658	−0.8542	0.0253
*SIRT3*	G5E521	sirtuin 3	11	333	−0.3330	0.0315
*CYP4A11*	F1ME58	cytochrome P450, family 4, subfamily A, polypeptide 11	3	514	−0.2852	0.0396
*PLCB2*	E1B7M6	Phospholipase C beta 2	10	1171	0.3344	0.0270

### Validation of DEGs and DEPs

To validate the accuracy of the DEGs detected by RNA-seq analysis, we used real-time reverse transcription-quantitative polymerase chain reaction (RT-qPCR) to evaluate the expression levels of eight DEGs: *PPARGC1A*, *DDIT4*, suppressor of *SOCS1*, solute carrier family 22 member 1 [*SLC22A1*], *HNF4A*, *PDGFA*, syntabulin [*SYBU*], and *MAPK9*. The expression levels of these genes in each group are shown in **Figure S6**. The eight genes selected were differentially expressed among the HP vs LP comparison group, and the RNA-seq data were concordant with those obtained by RT-qPCR (**Figure S6**).

The PRM assay was used to confirm the identity of several DEPs identified in the TMT analysis. Parallel reaction monitoring (PRM) technology uses a quadruple mass analyzer to selectively detect target proteins and target peptides. This technology has higher specificity and sensitivity than selected reaction monitoring (SRM) technology. As this assay requires the signature peptide of the target protein to be unique, we only selected proteins with a unique signature peptide sequence for the PRM analysis. Five DEPs (up-regulated:carnitine *CPT2*; down-regulated: Cytochrome [cyt b, *RDH13*, *CYP4A11*, and *SLC27A2*) were selected for the PRM analysis (**Figure S7**).

### Integrated Analysis of DEGs and DEPs From TMT and RNA-Seq Data

The Pearson correlation coefficient for the log2 function of HP vs. LP was 0.31, indicating that mRNA and protein levels were only partially correlated overall. Only *SLC22A1* and Heat shock protein family A member 13 [*HSPA13*] were identified as both DEGs and DEPs. On the basis of these results, we propose that post-transcriptional regulatory activity contributes to milk fat lipid anabolism.

### Integrated Analysis of DEGs, DEPs, and Animal QTLdb

We integrated DEGs and QTL for milk production traits from the QTLdb database that were detected either by QTL mapping studies or genome‐wide association studies (GWAS) by comparing their chromosome positions in order to gain further insight into the association between DEGs and milk fat traits. For QTL detected by QTL mapping studies, only those with a confidence interval less than 1 Mb were considered as a QTL region; for those identified by GWAS, the 200 kb up-/downstream of significant single nucleotide polymorphisms (SNPs) were defined as a QTL region. Among the DEGs and DEPs, 199 genes regions were located within or overlapped with QTL regions (**Figure S5**).

## Discussion

FAs in milk originate from two sources: some are synthesized *de novo* by mammary epithelial cells (MECs), including nearly all short (C4–C8) and middle chain (C10–C14) FAs and half of C16 FAs; the remaining C16 FAs and LCFAs (>C16) are obtained by MECs directly from the blood.

After rumen fermentation, digestive tract absorption, liver metabolism, and so on, compounds such as acetic acid, β-hydroxybutyric acid (BHBA), free (F) FAs, etc. from the absorption and conversion of dietary nutrients are used in the mammary gland to synthesize milk fat. The metabolism, transformation, and utilization of these precursors in the body directly affect milk fat content. In addition, acetic acid, BHBA, and FFAs act as signaling molecules to modulate lipid synthesis through a feedback mechanism in the liver and adipose tissue.

The lactation process of dairy cows has periodicity and can be divided into early non-lactating period, late non-lactating period, early lactation period, peak lactation period, middle lactation period, and late lactation period. The peak lactation period occurs 6–8 weeks after delivery. After the peak period to 30–35 weeks after delivery, it is the middle of lactation, and the milk yield in the medium term is slightly lower than that in the early stage; however, the milk components are relatively stable.

In this study, we identified genes associated with milk fat and milk FA production by examining the transcriptome and proteome profiles of liver tissue samples from Chinese Holstein cows with extremely high or low milk fat percentage. A comparative analysis revealed 321 DEGs and 76 DEPs; 8 DEGs of *PPARGC1A*, *DDIT4*, *SOCS1*, *SLC22A1*, *HNF4A*, *PDGFA*, *SYBU*, and *MAPK9* and 5 DEPs of *CPT2*, *cytb*, *RDH13*, *CYP4A11*, and *SLC27A2* were verified by RT-qPCR and PRM, respectively, and the results were consistent with the previous experiments, confirming the reliability of this multi-omics study. Some of the genes with known roles in milk production such as *DGAT1* (Grisart et al., 2004), growth hormone receptor[*GHR*] (Blott et al., 2003), and stearoyl-CoA desaturase[*SCD*] (Kinsella, 1972) did not differ between the two groups. It is likely that factors whose expression differed significantly between HP and LP cows have been fixed through long-term genetic selection. In particular, *SLC22A1* and *HSPA13* were identified as both DEGs and DEPs. A functional enrichment analysis identified for the first time 22 DEGs (*SLC22A1*, *MAPK9*, *PPARGC1A*, *FOXO1*, *SOCS1*, *SOCS2*, *CREB1*, *HNF4A*, *HNF4G*, *GADD45A*, *DUSP1*, *PDGF*, *SYBU*, *DDIT4*, BMP and activin membrane bound inhibitor [*BAMBI*], methylenetetrahydrofolate reductase [*MTHFR*], *SLC27A2*, *PCK1*, *CPT2*, *SIRT3*, *CYP4A11*, and *PLCB2*) as candidate genes that regulate milk fat synthesis, transport, and metabolism.

### DEGs for Milk Fat Traits

*MAPK9* encodes a member of the MAPK family. These proteins act as an integration point for multiple biochemical signals and are involved in a variety of cellular processes, including cell proliferation and differentiation, transcriptional regulation, and development. A previous study indicated that *MAPK9* is implicated in the response to intramammary challenge and negative energy balance in cows (Moyes et al., 2010). *MAPK9* is important in the proposed network of milk fat synthesis that includes encompassing MAPK [c-Jun N-terminal kinase (JNK)] and insulin signaling and insulin resistance (Liang et al., 2017). It is likely that the high expression of *MAPK9* is involved in integrating insulin production, insulin resistance, and MAPK and prolactin production signaling to increase lipid synthesis in the liver. *FOXO1* belongs to the forkhead family of transcription factors that are characterized by a distinct forkhead domain. The FOXO1/Akt pathway plays a critical role in gluconeogenesis in the liver (Yang et al., 2018). A previous transcriptome analysis of the liver suggested that *FOXO1* influences milk fat synthesis (Jacometo et al., 2016). In addition, the low expression levels of *FOXO1* suggested it may activate insulin production and insulin resistance signaling to enhance glucose and lipid metabolism in the liver. Suppressor of cytokine signaling (SOCS) family genes such as *SOCS1* and *SOCS2* encode signal transducer and activator of transcription (STAT)-induced STAT inhibitor proteins, which are cytokine-inducible negative regulators of cytokine signaling. A previous study reported several SNPs near the *SOCS1*, *SOCS3*, *SOCS5*, and *SOCS7* genes that were significantly associated with protein yield (Arun et al., 2015), suggesting that *SOCS1* and *SOCS2* interact with other genes to influence milk production and composition. CREB1 protein is phosphorylated by several protein kinases and induces gene transcription in response to hormonal stimulation *via* the cAMP pathway, leading to the regulation of lipid metabolism (Ikoma-Seki et al., 2015; Mucunguzi et al., 2017). *HNF4G* encodes HNF4γ, a nuclear transcription factor that binds DNA as a homodimer to control the expression of HNF1α, a transcription factor that regulates hepatic gene expression. *HNF4G* may also play a role in intramuscular fat deposition in beef cattle (Ramayo-Caldas et al., 2014), while the paralog *HNF4A* negatively regulates cholesterol metabolism and bile acid synthesis in the liver (Shirpoor et al., 2018). In the present study, the expression abundance of *HNF4G* and *HNF4A* in HP (2,881 and 11,382 reads) was threefold and twofold higher than LP groups (642 and 4,804 reads), respectively, revealing its high expression and importance in high milk fat percentage groups. *GADD45A* is up-regulated under stressful growth arrest conditions and by treatment with DNA-damaging agents. The protein encoded by this gene activates p38/JNK signaling *via* MEKK4/MTK1 kinase, which is known to regulate fat deposition in pork (Cho et al., 2015). In the present study, the expression of *DUSP1*was fivefold higher in the LP group (13,073 reads) than in the HP group (2,380 reads), suggesting that high expression levels of *DUSP1* modulate lipid metabolism and synthesis (Nukitrangsan et al., 2011). *PDGF* belongs to the same protein family as vascular endothelial growth factors, which play an essential role in the regulation of embryonic development; cell proliferation, migration, and survival; and chemotaxis. PDGF is also involved in the synthesis of monounsaturated FAs in cells (De Brachene et al., 2017). *SYBU* encodes a microtubule-associated protein that mediates anterograde transport of vesicles to neuronal processes. *SYBU* is phosphorylated by exchange protein directly activated by cAMP (Epac)2 agonist 8-pCPT-2′-O-Me-cAMP (Ying et al., 2012). The findings of the present study suggest that a high expression level of *SYBU* is an effector of Epac2 that contributes to cAMP-induced insulin secretion as well as milk production and composition. *DDIT4* regulates cell growth, proliferation, and survival by suppressing the activity of mTOR complex 1, which is involved in the response to changes in cellular energy level and stress. *DDIT4* has been proposed to be a negative regulator of cell proliferation and cell growth in goat, thereby affecting the synthesis of milk fat (Crisà et al., 2016). *BAMBI* stimulates adipogenesis by suppressing carboxypeptidase A4—a negative regulator of adipogenesis that modulates local and systemic insulin sensitivity—through interactions with genes known to regulate milk production and composition (He et al., 2016). *MTHFR* catalyzes the conversion of 5,10-methylenetetrahydrofolate to 5-methyltetrahydrofolate (nicotinamide adenine dinucleotide phosphate), a co-substrate for homocysteine remethylation to methionine. *MTHFR* gene deficiency may enhance liver injury by altering methylation capacity, inflammation, and lipid metabolism (Leclerc et al., 2018).

### DEPs for Milk Fat Traits

FAs entering the liver are mainly derived from FFAs produced by body fat mobilization. Triglyceride (TG) in adipose tissue is hydrolyzed into FFAs and glycerol by hormone-sensitive lipase and released into the blood. FFAs form a complex with albumin that is absorbed and utilized by the liver; FA transport protein (FATP)2 encoded by *SLC27A2* is a transmembrane protein transporter involved in this uptake. FATP2 has very long-chain acyl-CoA synthetase activity and converts free (F) LCFAs into fatty acyl-CoA esters. Interestingly, FATP2 expression was 1.28-fold higher in LP compared to HP cows. This may be related to lipid accumulation in the liver, which reduces milk fat precursor production in the liver. FATP2 overexpression in the liver is related to hepatic steatosis (Krammer et al., 2011), which reflects increased accumulation of lipids (mainly TG) in hepatocytes. Although FATP2 promotes the uptake of LCFAs, the esterification rate of LCFAs in the liver is higher than its decomposition rate and the rate of very (V)LDL transport to remove TGs, which thus accumulates as the lipid concentration in the cytoplasm decreases. Indeed, high *SLC27A2* transcript levels in blood cells are associated with lower TG levels (Sanchez et al., 2012). However, platelet glycoprotein 4 [CD36]—another LCFA transporter—showed a tendency (albeit non-significant) towards overexpression in HP, suggesting that LCFA uptake occurs *via* distinct mechanisms. PCK1 catalyzes the irreversible formation of phosphoenolpyruvate from oxaloacetate in gluconeogenesis. In non-ruminant animals, *PCK1* expression is induced by starvation and decreases during feeding. It is repressed by insulin and induced by glucagon and glucocorticoids (Hanson and Reshef, 1997). However, in ruminants its expression is not related to feed restriction but is induced by increased feed intake and monensin feeding, leading to increased ruminal propionate production (Greenfield et al., 2000; Velez and Donkin, 2005; Karcher et al., 2007). *PCK1* promoter activity is linearly induced by propionate in bovine (Zhang et al., 2016). In the present study, the *PCK1* protein level was higher in the LP than in the HP group, indicating a higher rate of gluconeogenesis in the liver. Increased utilization of hepatic lactolipid synthesis precursors such as propionic acid to generate glucose enhances hepatic FA oxidation to CO_2_, reflecting a redistribution of lactolipid precursors in the liver. *PCK1* is also involved in glyceroneogenesis, which catalyzes the production of glycerol-3-phosphate for FA esterification (Beale et al., 2007; Hosseini et al., 2015). Thus, the up-regulation of PCK1 in LP may be associated with TG accumulation in the liver. The increased level of acyl-CoA oxidase1—an enzyme involved in FA β-oxidation that stimulates ATP production to support gluconeogenesis and prevent lipid esterification and accumulation in the liver (Aoyama et al., 1994)—in LP suggests that lipid balance is regulated *via* modulation of glucose metabolism. Feeding strategies that increase rumen propionate production and thus induce *PCK1* expression are often used to meet increased glucose requirements and reduce the effects of fatty liver during early lactation in cows (Zhang et al., 2016). Consistent with our findings, cows with high liver fat content showed elevated expression of hepatic gluconeogenesis genes (Hammon et al., 2009), suggesting a higher gluconeogenic capacity in the liver. PPARγ is a known pro-adipogenic factor. We found here that the family with sequence similarity (FAM)120A—also known as bovine constitutive coactivator of PPARγ-like protein 1—was highly expressed in the LP group. PPAR signaling promotes the expression of the FA oxidation-related genes *CYP4A11* and *CPT2*, which is consistent with our results. We speculate that liver FA oxidation capacity is higher in the LP than in the HP group, leading to a reduction in milk fat synthesis precursors. β-Oxidation is the main pathway of FA catabolism. *CPT2* converts acylcarnitine translocated to the mitochondrial matrix into acyl-CoA and free carnitine and is a rate-limiting enzyme for the transport of LCFAs into mitochondria for β-oxidation (Isackson et al., 2013). Silent mating type information regulation 2 homolog (Sirt3) regulates FA β-oxidation in the liver *via* AMPK and sterol regulatory element-binding protein1, promoting FA utilization and thereby preventing fat heterotopia (Kong et al., 2016). *CPT2* and *Sirt3* proteins were highly expressed in the LP group, indicating enhanced FA β-oxidation. In addition to β-oxidation, microsomal ω-oxidation mediated by cytochrome P450 enzymes played a key role in lipid synthesis and lipid accumulation (Hardwick, 2008). Through preferential hydroxylation of FA chain terminal methyl groups, CYP4A/4F subfamily members eliminate potentially toxic, excess non-esterified FFAs that could disrupt mitochondrial function and inhibit ATP synthesis (Sanders et al., 2006; Weinberg, 2006; Hsu et al., 2007). Thus, the increase in *CPY4A11* protein expression in the LP group indicates that the ability of liver cells to oxidize non-esterified FAs was enhanced. ω-Oxidation is increased in non-alcoholic fatty liver disease (Kohjima et al., 2007). In the LP group, increased FA oxidation may induce lipid accumulation in the liver and suppress the synthesis of milk fat, although the specific mechanisms remain to be determined. 20-Hydroxyeicosatetraenoic acid (20-HETE) is a product of arachidonic acid that is hydroxylated by *CYP4A11* catalysis, which can stimulate the production of superoxide and inflammatory cytokines, inhibit endogenous nitric oxide synthase, and promote oxidative stress (Lasker et al., 2000; Singh et al., 2007; Cheng et al., 2008; Ishizuka et al., 2008). 20-HETE metabolites can enhance the hypertrophy of mature inflamed adipocytes in populations of mesenchymal stem cells undergoing adipogenic differentiation through pro-adipogenic effects (Kim et al., 2013). 20-HETE and its metabolites also activate PPARγ (Ashley et al., 2002; Fang et al., 2007) to induce adipogenesis. The up-regulation of FAM120A protein in the LP group suggests that 20-HETE plays a role in regulating the metabolism of lipid precursors in the liver, with the elevation in the *CYP4A11* protein level reflecting activation of downstream signaling. The activation of *PLCB2* is thought to play an important role in the regulation of glucose-induced insulin secretion (Zawalich et al., 1997). The observed up-regulation of *PLCB2* in the HP group may be responsible for the down-regulation of *PCK1* and *CYP4A11*, which are inhibited by increased insulin secretion in the liver (Lobato et al., 1985; Gainer et al., 2005).

### Integrated Analysis of DEGs and DEPs

Only *SLC22A1* and *HSPA13* were identified in DEGs and DEPs, and their trends were consistent: *SLC22A1* was significantly down-regulated in the HP group, while *HSPA13* was significantly up-regulated in the HP group, both at the transcriptional and proteomic levels. *SLC22A1* is one of three similar cation transporter genes located in a cluster on chromosome 9. Polyspecific organic cation transporters in the liver, kidney, intestine, and other organs are critical for the elimination of endogenous small organic cations. The encoded protein contains 12 putative transmembrane domains and is a plasma integral membrane protein. A previous study showed that loss of OCT1 (*SLC22A1*) caused an increase in the ratio of AMP to ATP, activated the energy sensor AMP-activated kinase (AMPK), and substantially reduced triglyceride (TG) levels in livers from healthy mice (Ligong et al., 2014). An important paralog of this gene is *SLC22A7*, which is involved in milk fat synthesis in the liver (Liang et al., 2017). *HSPA13*, A member of the HSP 70 family, is mainly involved in stress-induced protective responses (Yan et al., 2015). Migdalska et al. (2012) found in the a mouse partial monosomy model for human chromosome 21q11.2-q21.1 that down-regulated expression of HSPA13 resulted in severe liver fatty changes and thickened subcutaneous fat when mice were fed a high-fat diet (HFD), then suspected that this gene may regulate fat deposition. This is consistent with our study finding that the expression level of *HSPA13* in LP group was significantly lower than that in HP group, both at the transcription level and protein level. In addition, HSPA13 is localized in microsomes (Kampinga et al., 2009) and may be one of the upstream factors for further inducing ω-oxidation in LP group, but its role in CPY4A11, FAM120A, and other ω-oxidation-related proteins in this study needs to be further studied. We found that 53 genes were found in DEGs and non-significantly different proteins, and 81 genes were found in non-significantly different genes and DEPs, which indicated the imbalance between proteomic and transcriptome data. There are two possible reasons: First, post-translational modifications (PTMs) play a vital role in the structure, activity and function of protein, and the activity of protein suggests that it is not the level of its expression that determines its function. Second, the currently multi-omics technologies still need to be improved, which not only requires more accurate quantitative technologies but also needs to detect the PTM level of proteins, which is the focus of our next research.

## Conclusions

In this study, we identified genes and proteins involved in the regulation of milk composition and production in Chinese Holstein cows. Through RNA-seq and TMT analyses of liver tissue we generated transcriptomic and proteomic profiles that revealed the regulatory relationships between DEGs and DEPs as well as several key candidate regulatory molecules (*SLC22A1*, *MAPK9*, *PPARGC1A*, *FOXO1*, *SOCS1*, *SOCS2*, *CREB1*, *HNF4A*, *HNF4G*, *GADD45A*, *DUSP1*, *PDGF*, *SYBU*, *DDIT4*, *BAMBI*, *MTHFR*, *SLC27A2*, *PCK1*, *CPT2*, *SIRT3*, *CYP4A11*, and *PLCB2*) and pathways (insulin, insulin resistance, PI3K/Akt, MAPK, prolactin, mTOR, and PPAR) associated with milk fat synthesis. These results can serve as a basis for breeding Holstein cows that produce milk with abundant essential fats, proteins, and other nutrients.

## Ethics Statement

All procedures involving the handling of experimental animals were conducted in accordance with and were approved by the Animal Welfare Committee of China Agricultural University (permit no. DK996).

## Author Contributions

SZ conceived and designed the study and revised the manuscript. CZ and DS performed the phenotype collection, sample collection, and data analysis and drafted the manuscript. CL participated in the experimental design and drafted the manuscript. SL, WC, MC, and SS participated in sample collection. All authors have read and approved the final manuscript.

## Funding

This work is supported by the 863 project (2013AA102504), the National Science and Technology Programs of China (2011BAD28B02), National Key Technologies R & D Program (2012BAD12B01), Beijing Dairy Industry Innovation Team, China Agricultural Research System (CARS-36), and Xinjiang Province Key Technology Integration and Demonstration Program (201230116). We are deeply grateful to all donors who participated in this program.

## Conflict of Interest Statement

The authors declare that the research was conducted in the absence of any commercial or financial relationships that could be construed as a potential conflict of interest.
