# Supplementary figures and images for: Comparative Transcriptomic and Proteomic Analyses Identify Key Genes Associated With Milk Fat Traits in Chinese Holstein Cows
Source: Front Genet. 2019 Aug 13;10:672. doi: 10.3389/fgene.2019.00672 (PMC6700372; doi:10.3389/fgene.2019.00672)

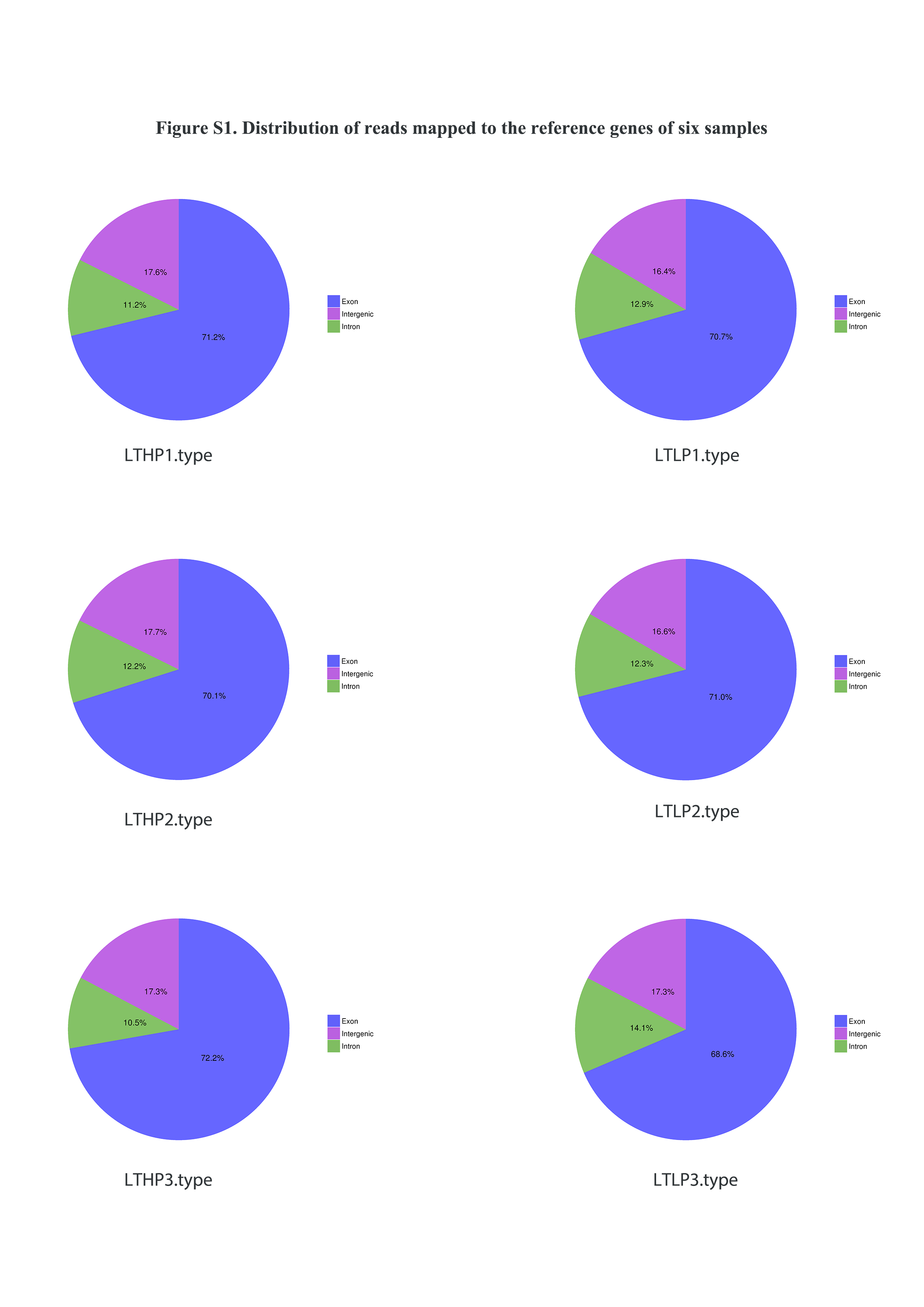

Supplement: Supplementary file 1 [file Image_1.png]

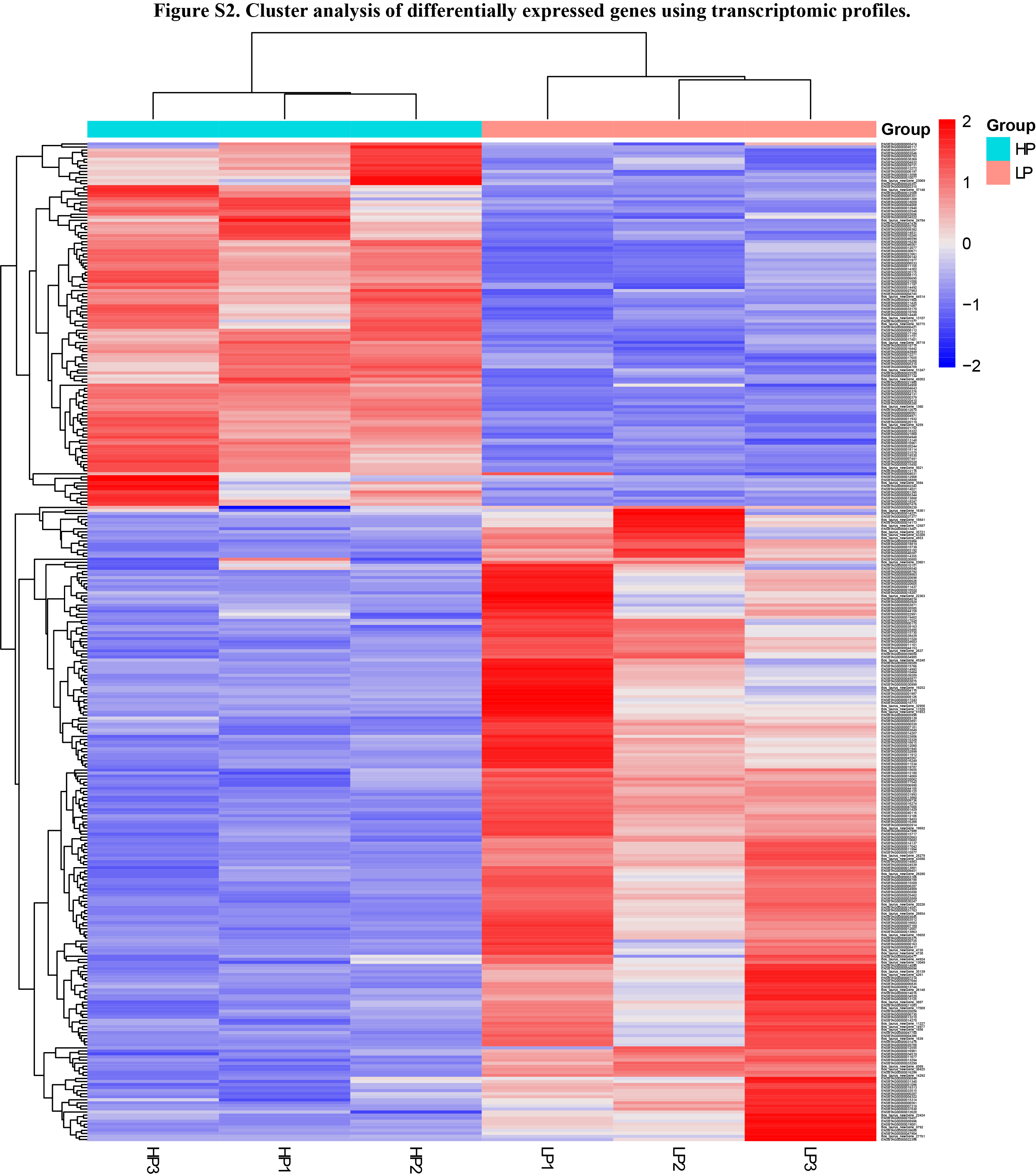

Supplement: Supplementary file 2 [file Image_2.png]

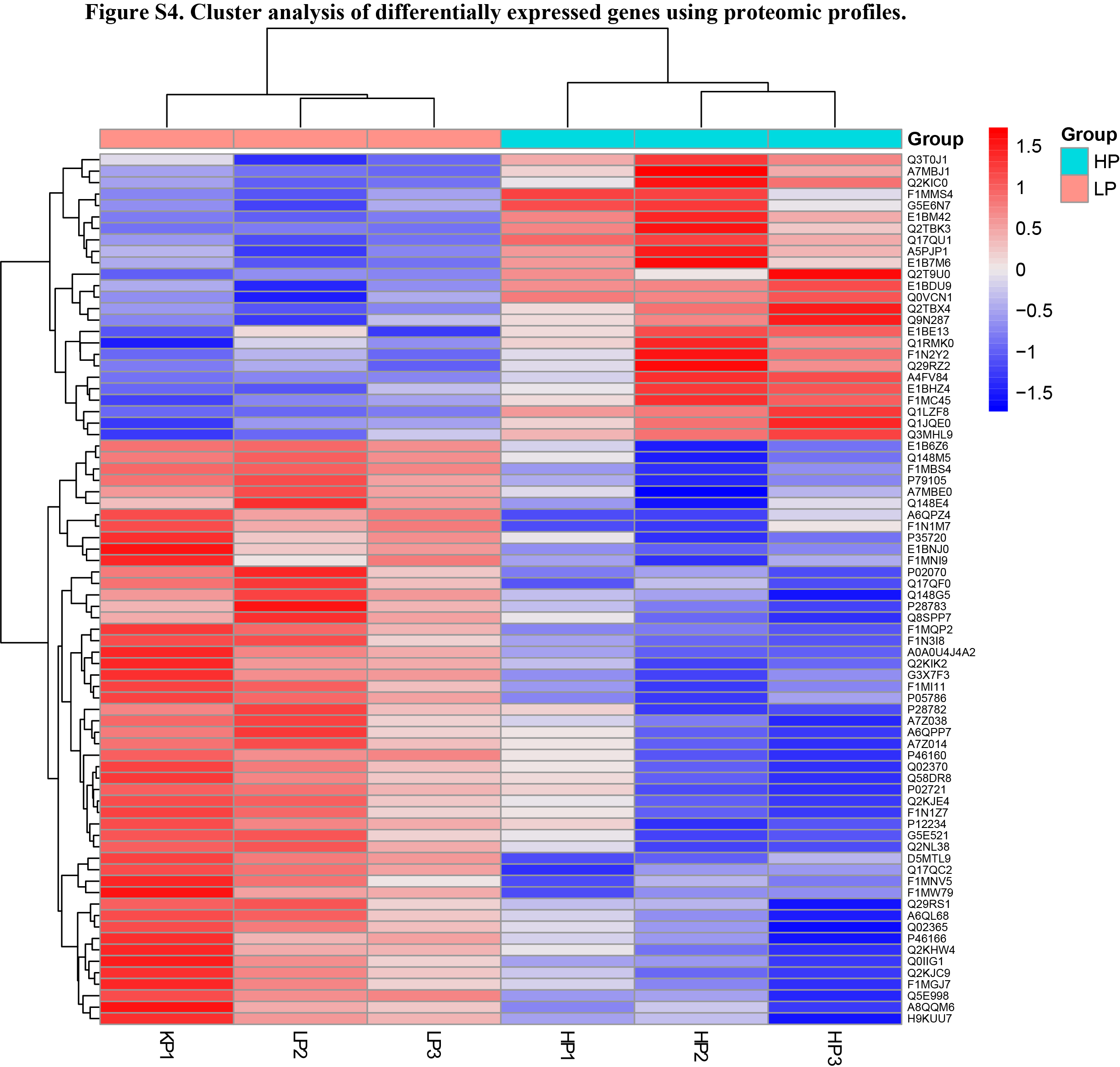

Supplement: Supplementary file 4 [file Image_4.png]

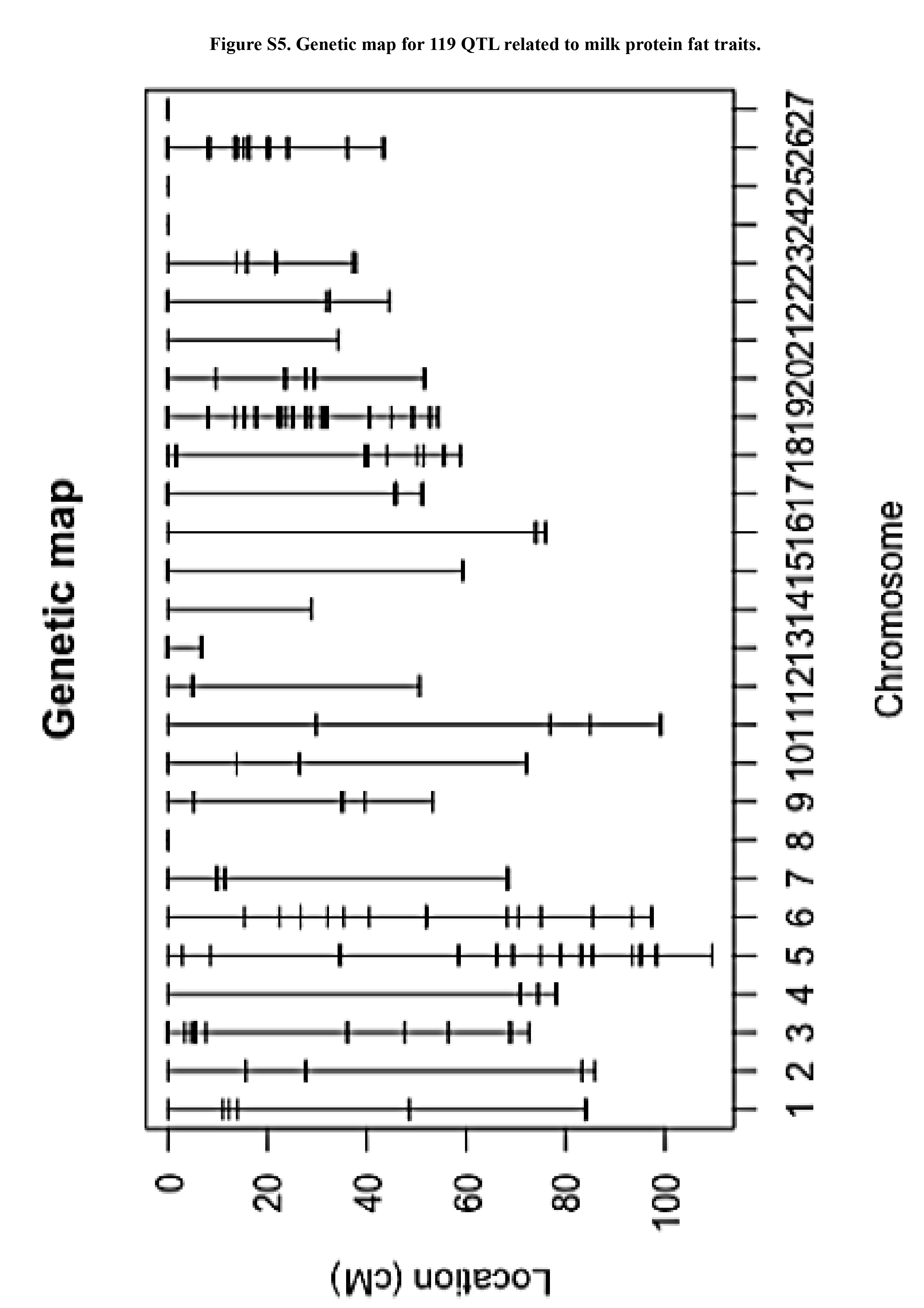

Supplement: Supplementary file 5 [file Image_5.png]
