# Supplementary material for: Comparative Transcriptomic and Proteomic Analyses Identify Key Genes Associated With Milk Fat Traits in Chinese Holstein Cows
Source: Front Genet. 2019 Aug 13;10:672. doi: 10.3389/fgene.2019.00672 (PMC6700372; doi:10.3389/fgene.2019.00672)

Figure S3(a-d). Overview of protein identification information. (a) Basic information on protein identification. (b) Distribution of the proteins identified according to molecular weight. (c) Protein coverage by the peptides identified. (d) Distribution of proteins containing different numbers of identified peptides.

Fig. S3 (a) Basic information on protein identification.

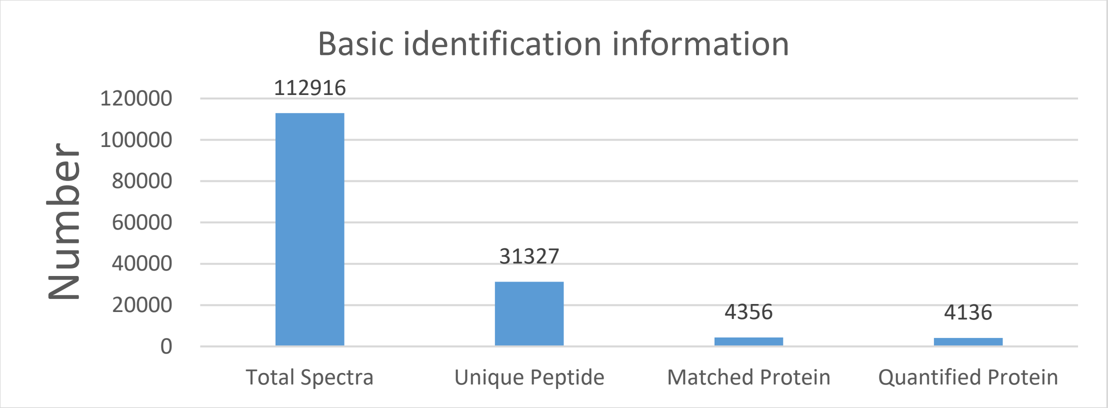

Fig.S3 (b) Distribution of the proteins identified according to molecular weight

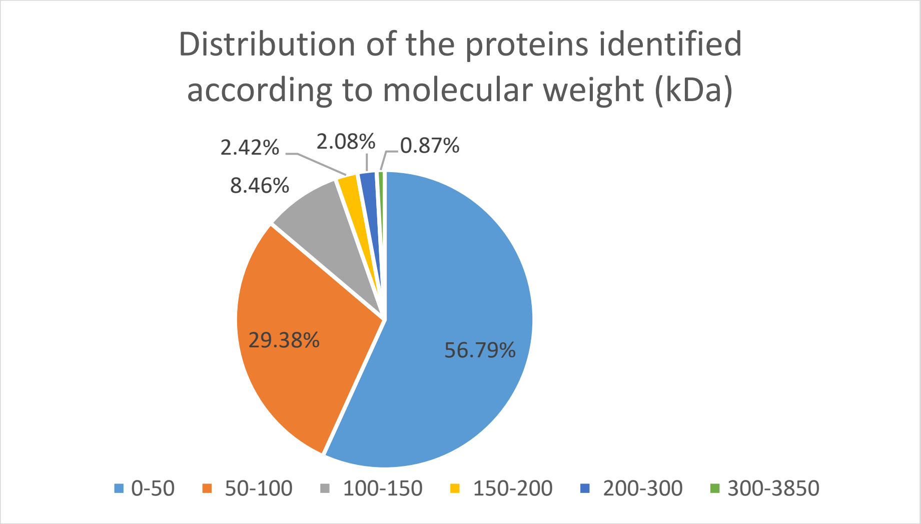

Fig.S3 (c) Protein coverage by the peptides identified

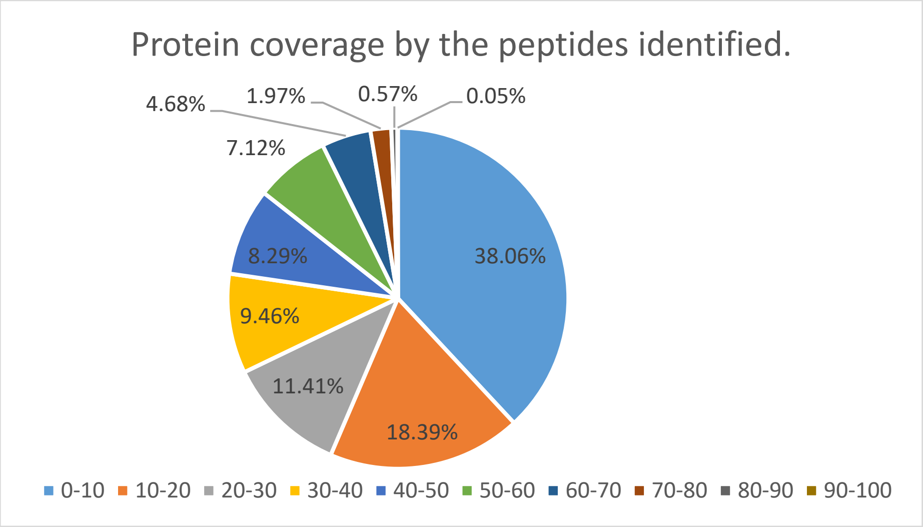

Fig.S3 (d) Distribution of proteins containing different numbers of identified peptides.

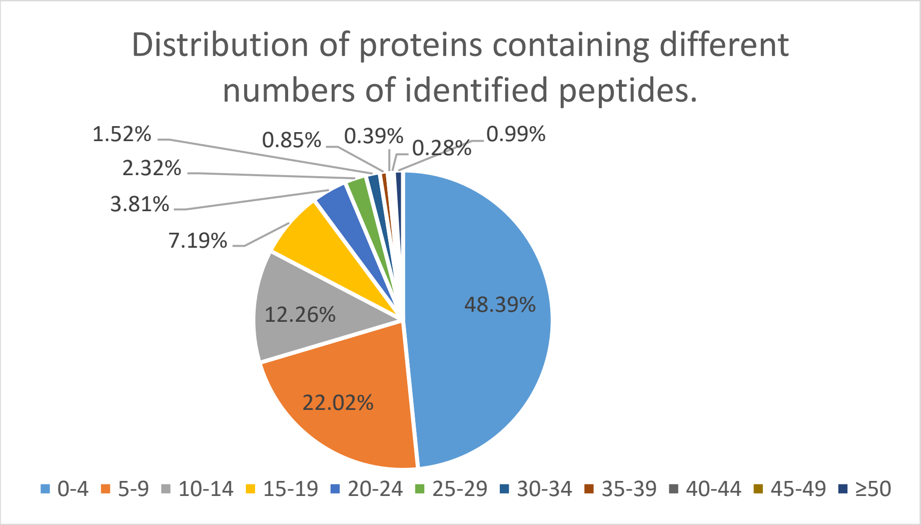

Supplement: Supplementary file 3 [file Image_3.pdf]
