# Supplementary material for: Comparative Transcriptomic and Proteomic Analyses Identify Key Genes Associated With Milk Fat Traits in Chinese Holstein Cows
Source: Front Genet. 2019 Aug 13;10:672. doi: 10.3389/fgene.2019.00672 (PMC6700372; doi:10.3389/fgene.2019.00672)

**Figure S6. The comparisons of expression abundance of 8 randomly differentially expressed genes between qRT-PCR and RNA-Seq**

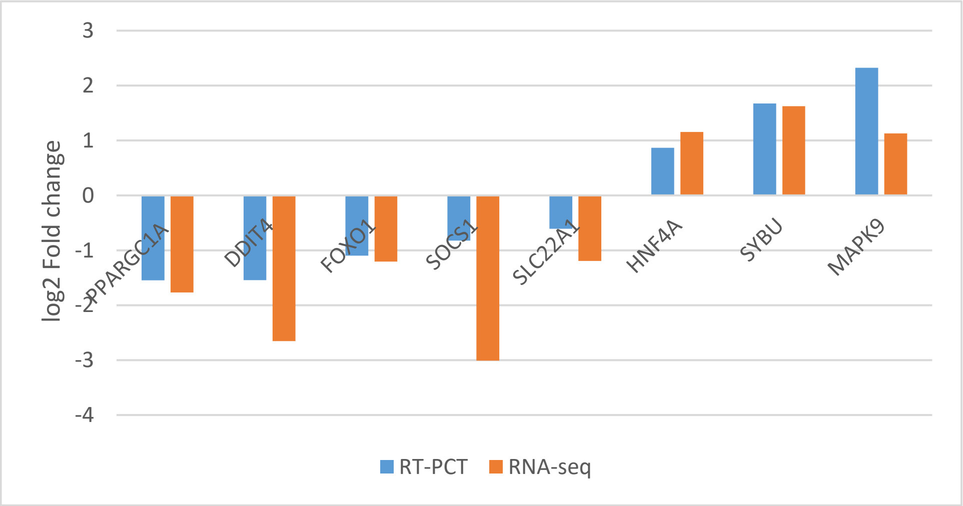

Supplement: Supplementary file 6 [file Image_6.pdf]
