# Supplementary material for: Comparative Transcriptomic and Proteomic Analyses Identify Key Genes Associated With Milk Fat Traits in Chinese Holstein Cows
Source: Front Genet. 2019 Aug 13;10:672. doi: 10.3389/fgene.2019.00672 (PMC6700372; doi:10.3389/fgene.2019.00672)

Figure S7. The comparisons of expression level of 5 randomly differentially expressed proteins between PRM and TMT

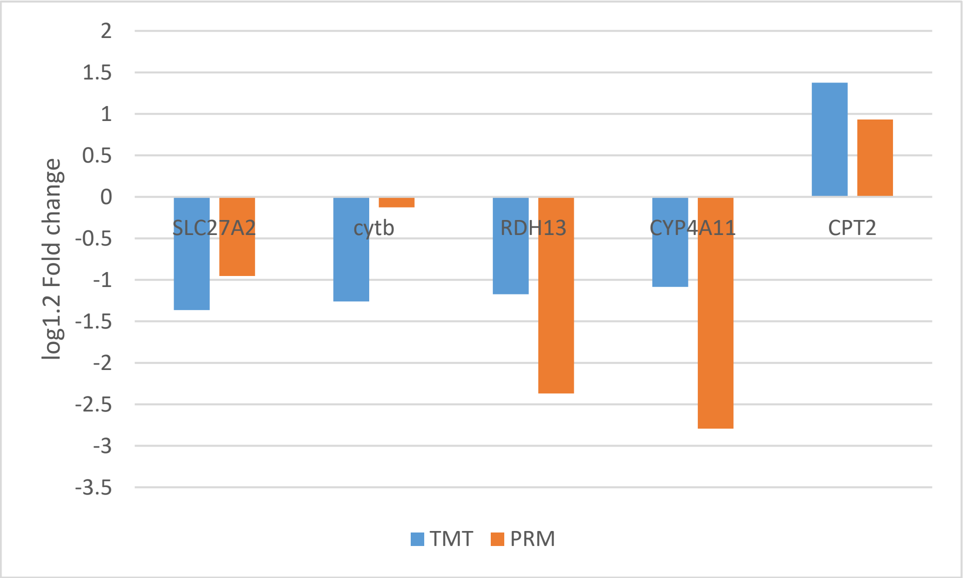

Supplement: Supplementary file 7 [file Image_7.pdf]
