# Supplementary material for: Comparative Transcriptomic and Proteomic Analyses Identify Key Genes Associated With Milk Fat Traits in Chinese Holstein Cows
Source: Front Genet. 2019 Aug 13;10:672. doi: 10.3389/fgene.2019.00672 (PMC6700372; doi:10.3389/fgene.2019.00672)
Supplement: Supplementary file 8 [file Table_1.docx]

### Table S1 The phenotype information of six Chinese Holstein cattle

| Group | Sample ID | Parity | Days in milk | Fat percentage | Protein percentage |
| --- | --- | --- | --- | --- | --- |
| HP | HP 1 | 2 | 232 | 3.74% | 3.47% |
| HP | HP 2 | 2 | 382 | 3.90% | 4.07% |
| HP | HP 3 | 2 | 230 | 3.77% | 3.73% |
| LP | LP 1 | 2 | 233 | 3.12% | 3.14% |
| LP | LP 2 | 2 | 154 | 2.85% | 3.04% |
| LP | LP 3 | 2 | 157 | 3.20% | 3.21 |
